# Hematopoietic stem cell transplantation and cellular therapies for autoimmune diseases: overview and future considerations from the Autoimmune Diseases Working Party (ADWP) of the European Society for Blood and Marrow Transplantation (EBMT)

**DOI:** 10.1038/s41409-022-01702-w

**Published:** 2022-05-16

**Authors:** Tobias Alexander, Raffaella Greco

**Affiliations:** 1 Department of Rheumatology and Clinical Immunology, Corporate Member of Freie Universität Berlin, Humboldt-Universität zu Berlin, Berlin Institute of Health, Charité—Universitätsmedizin Berlin, Berlin, Germany; 2 Unit of Hematology and Bone Marrow Transplantation, IRCCS San Raffaele Scientific Institute, Vita-Salute San Raffaele University, Milan, Italy

**Keywords:** Autoimmune diseases, Immunotherapy

## Abstract

Autoimmune diseases (ADs) represent a heterogenous group of complex diseases with increasing incidence in Western countries and are a major cause of morbidity. Hematopoietic stem cell transplantation (HSCT) has evolved over the last 25 years as a specific treatment for patients with severe ADs, through eradication of the pathogenic immunologic memory and profound immune renewal. HSCT for ADs is recently facing a unique developmental phase across transplant centers. This review provides a comprehensive overview of the recent evidence and developments in the area, including fundamentals of preclinical research, clinical studies in neurologic, rheumatologic and gastroenterologic diseases, which represent major indications at present, along with evidence of HSCT for rarer indications. Moreover, we describe the interwoven challenges of delivering more advanced cellular therapies, exploiting mesenchymal stem cells, regulatory T cells and potentially CAR-T cell therapies, in patients affected by ADs. Overall, we discuss past and current indications, efficacy, associated risks and benefits, and future directions of HSCT and advanced cellular therapies in the treatment of severe/refractory ADs, integrating the available literature with European Society for Blood and Marrow Transplantation (EBMT) registry data.

## Introduction

Over the last 25 years, hematopoietic stem cell transplantation (HSCT) has been increasingly used to treat patients affected by severe and refractory autoimmune diseases (ADs) [1]. The majority of such patients have chronic diseases, which impact on quality-of-life and can shorten life expectancy but are rarely life-threatening in the short-term. Autologous and allogeneic HSCT are performed in this population after a careful balance of benefits and risks, and consideration of alternative treatment options [2, 3], with the aim to ablate the aberrant immune system and reconstitute one more tolerant to self-antigens, inducing a complete and stable remission from disease activity [4].

**Fig. 1:**
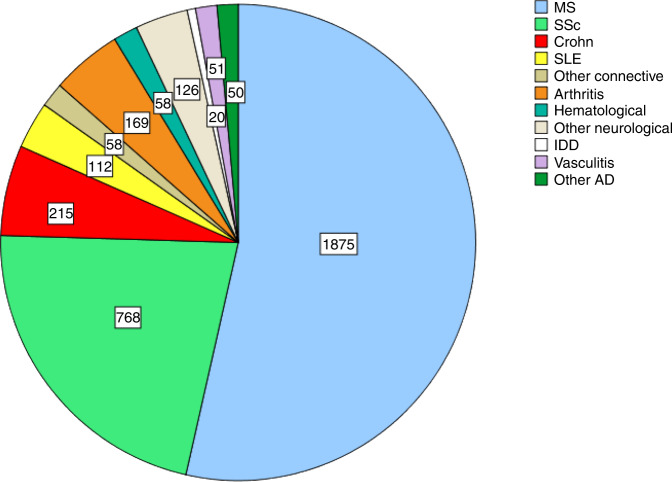
EBMT-ADWP Registry data: autologous HSCT for ADs (1994–2021, *n* = 3502). Autologous transplants for ADs reported to the EBMT-ADWP data registry, from 1994 through 2021. Major indications for autologous HSCT are multiple sclerosis, systemic sclerosis, and Crohn’s disease. AD autoimmune disease, ADWP Autoimmune Diseases Working Party, EBMT European Society for Blood and Marrow Transplantation, HSCT hematopoietic stem cell transplantation, IDD insulin-dependent diabetes mellitus, MS multiple sclerosis, SSc systemic sclerosis, SLE systemic lupus erythematosus.

**Fig. 2:**
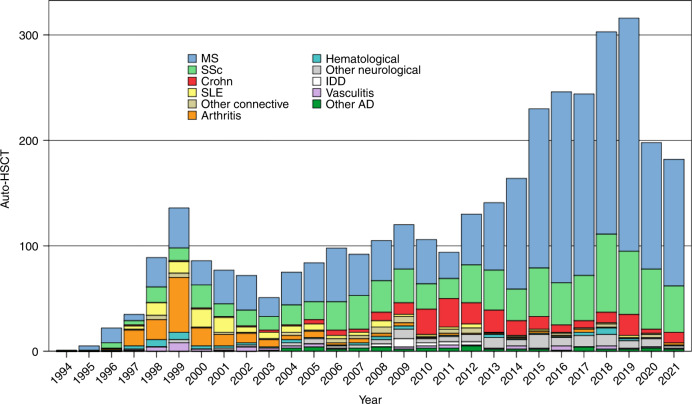
Autologous HSCT for AD, reported to the EBMT-ADWP Registry data, per year and indication. Number of autologous HSCT reported per year and indication, from 1994 through 2021 (*n* = 3502). AD autoimmune disease, ADWP Autoimmune Diseases Working Party, EBMT European Society for Blood and Marrow Transplantation, HSCT hematopoietic stem cell transplantation, IDD insulin-dependent diabetes mellitus, MS multiple sclerosis, SSc systemic sclerosis, SLE systemic lupus erythematosus.

Recent evidence base, as developed across the international scientific research communities, explored HSCT as single one-off procedure to treat a variety of severe Ads [5]. Multiple sclerosis (MS) and systemic sclerosis (SSc) cover around 80% of transplants performed for ADs (Figs. 1, 2), where HSCT has become an integral and standard-of-care part of treatment algorithms [6, 7].

An analysis of trends across all ADs over the first 20 years of activity reflected the growth across indications, improvements in outcomes related to experience and center size, with some support for benefit of accreditation and economic factors [8]. A multidisciplinary approach is key in this field. The EBMT Autoimmune Diseases Working Party (ADWP) is central to bringing together HSCT and disease specialist communities. The AD section of the EBMT Registry is the largest database of its kind worldwide, reporting more than 3700 transplants.

In this review, we discuss past and current indications, efficacy, associated risks and benefits, and future directions of HSCT and advanced cellular therapies in the treatment of severe/refractory ADs, integrating the available literature with EBMT registry data. Moreover, we offer a thorough insight into the pathophysiology and management of ADs, and provide a general guidance on the entire transplant process, from the biological rationale and preclinical models to clinical results and recommendations.

## Biology of immune cells during aging, autoimmunity, and stem cell transplantation

Despite the clinical heterogeneity of ADs, many of them share common genetic and molecular patterns leading to a loss of tolerance and chronic immunologic self-reactivity [9]. On a cellular level, this is reflected by profound age-associated changes of B cells, such as decreased B-cell differentiation in the bone marrow [10], redistribution of B-cell subsets in the periphery with a significant increase of proinflammatory (CD19^+^CD27^−^IgD^−^) double-negative B cells [11], and decreased expression of molecules involved in immunoglobulin class switching and somatic hypermutation, leading to decreased repertoire diversity and alterations of antibody responses [12]. Similarly, T cells undergo profound alterations during the aging process, ultimately resulting in immunosenescence [13], most importantly associated with thymic involution with reduced T-cell receptor repertoire diversity [14, 15]. In contrast, antibody-secreting memory plasma cells, which are most recognizable for their longevity [16], are stably maintained during aging [17], as their survival is controlled by dedicated survival niches in the bone marrow [18]. Importantly, long-lived plasma cells not only provide protective humoral immunity, they also contribute to the chronicity of various ADs by the persistent secretion of pathogenic antibodies [19], and thus, resemble novel therapeutic targets in autoimmunity [20]. The effect of HSCT on immunologic renewal has been the focus of many mechanistic studies. They collectively demonstrated that many of the age- or autoimmune-mediated immune disturbances may improve or even resolve [5]. For example, immune reconstitution studies indicated a predominant repopulation of CD27^−^IgD^+^ naïve B cells [21, 22], a stable output of recent thymic emigrants [23, 24], leading to a re-diversification of the receptor repertoire, and recurrence of both regulatory B and T cells in various Ads [25–28].

## General concepts and process of HSCT for ADs

The initial concept of using HSCT as a means of controlling severe ADs was based on animal experiments and case reports showing long remission of ‘coincidental’ AD alongside standard hematological HSCT indications [29, 30]. Autologous HSCT is largely adopted to provide rapid and sustained hematopoietic recovery, supporting regimens of high-dose cytotoxic chemotherapy with lymphodepleting serotherapy (most commonly anti-thymocyte globulin, ATG). Nowadays, due to the less invasive collection methods and more rapid resolution of neutropenia and thrombocytopenia, peripheral blood stem cells (PBSCs) have largely replaced bone marrow as source, using cryopreservation techniques. After mobilization with granulocyte-colony stimulating factor (G-CSF) with or without cyclophosphamide (Cy), the graft is collected by leukapheresis and may be manipulated to enrich hematopoietic stem cells (CD34 selection) [31] and remove immune cells (ex vivo T cell depletion), potentially removing self-reactive lymphocytes [5].

Different conditioning regimens may be administered before the reinfusion of the cells in patients affected by ADs. The overall intensity of the conditioning regimen [32] varies greatly according to disease indication, study protocol and center preference. The optimal intensity of the conditioning is still to be defined in ADs, due to wide clinical variability of patients and lack of direct comparative trials. The final intensity of the immunosuppression is affected by many factors (type of conditioning and inclusion/dosage of serotherapy, mobilization chemotherapy, CD34 selection as graft manipulation, and prior treatments), leading to consider more ‘treatment intensity’ rather than just ‘conditioning intensity’ in the context of Ads [2, 5]. Reduced intensity [33] conditioning regimen significantly reduce the risk of treatment-related morbidity such as infections, infertility, organ damage, as well as treatment-related mortality (TRM). Indeed, there was chronological improvement in HSCT outcomes (i.e., progression-free survival, relapse/progression, and TRM), strictly connected to the transplant center experience, patient selection and progress with supportive care [8].

HSCT exerts its therapeutic effect in ADs through various mechanisms, including the immunosuppressive conditioning regimen able to eradicate the autoreactive immunologic memory, and the regeneration and renewal of the immune system, leading to the re-induction of immune tolerance to rewire aberrant immune response toward self-antigens [26, 34]. Moreover, changes in the microbiome profile [35], which have been linked to various ADs, merit further investigations in the context of immune recovery. The development of an immunological balance allows long-term disease remission [36].

## Considerations for HSCT in neurological autoimmune diseases

Neurological ADs may affect the central and peripheral nervous systems, resulting in a variety of manifestations and symptoms. MS is currently the most frequent autoimmune disease for which HSCT has been used, accounting for 1875 patients reported in the EBMT registry (Fig. 1). Increasing evidence [5] support the use of autologous HSCT as highly effective therapeutic strategy for treatment-resistant inflammatory types of MS [33, 36–40], for which it can be currently regarded as a standard of care [6].

Remission of disease activity in patients with severe active MS undergoing autologous HSCT relies not only on the effect of high-dose chemotherapy and intense lymphodepletion, but also on the renewal [34] of the immune compartment. Initially conceived as an extreme “rescue” therapy in MS patients with a poor prognosis who have failed all other therapies, delivery of HSCT subsequently changed [41–47], including more patients in earlier inflammatory phases of the disease, with a gradual shift to a major predominance of relapsing remitting over progressive forms [8, 48]. This trend was also supported by the EBMT data [6].

Many patients, especially in Europe, have been conditioned with “intermediate intensity” conditioning regimens, such as BEAM (BCNU 300 mg/m^2^ on day −6, cytosine arabinoside, 200 mg/m^2^ and etoposide 200 mg/m^2^ day −5 to day −2, melphalan 140 mg/m^2^ day −1) plus rabbit ATG in dose range of 5–7.5 mg/kg, showing satisfactory toxicity/efficacy equipoise, even in the long-term follow-up [36]. Recently, low intensity regimens involving administration of Cy and ATG have been adopted, showing prolonged time to disease progression as compared to approved disease-modifying therapy (DMT) [33]. New studies are required to better define the optimal conditioning and solve this current regimen selection controversy. Importantly, autologous HSCT seems to offer clear advantage in terms of NEDA (no evidence of disease activity), showing rates of 66–93% compared with alemtuzumab, natalizumab or ocrelizumab [6].

In recent years, as clearly demonstrated by a marked decrease in TRM to 0.2%, better outcomes have been obtained owing to a growing experience in selecting the most appropriate patients to transplant, paralleled by advances in conditioning and support regimens [4, 8]. Despite the recent COVID-19 pandemic, the non-relapse mortality (NRM, defined as death for whatever cause, without ever experiencing relapse) remains stable around 1% from 2015 through all the 2020, according to the recent EBMT registry data.

Moreover, HSCT may be considered as “clinical option” in carefully selected patients affected by rare treatment-resistant neurological Ads [6]. Autologous HSCT is effective also in refractory and aggressive neuromyelitis optica [49, 50], chronic inflammatory demyelinating polyradiculoneuropathy [51], as well as myasthenia gravis [52], stiff-person syndrome disorder [53, 54], and anti-GAD (glutamic acid decarboxylase)-mediated encephalitis [55].

## Considerations for HSCT in rheumatic diseases

Although the areas of application of HSCT for rheumatic diseases have changed over the past 25 years, they still represent major indications with 1158 patients reported to the EBMT registry to date (Fig. 1). Initially, HSCT has been applied for rheumatoid arthritis and juvenile idiopathic arthritis, where studies demonstrated mixed results with high rates of persistent or relapse of disease activity within 6 months of transplant requiring continued DMT [56, 57], not justifying the further development of HSCT in these indications. Subsequently, HSCT has been utilized for systemic lupus erythematosus (SLE) with more than 300 patients being treated worldwide so far [5]. Data from large single-center experiences and multicenter trials indicate a disease-free survival of ~50–66% at 5 years despite discontinuation of immunosuppressive and other targeted therapies [21, 58–61]. Responding patients are usually free of clinical symptoms and may regain seronegativity for antinuclear antibodies, which is rarely seen under conventional therapies. Early use of HSCT also has the potential to protect against organ-failure and toxicity-related morbidity and improve quality-of-life [61]. The relapse rate is higher in patients receiving unmanipulated stem cell grafts and conditioning regimens without serotherapy [60], potentially reflecting the reactivation of autoimmune responses initiated by residing long-lived memory plasma cells, which reasonably should be in the focus of future targeted approaches complementing Cy/ATG-based regimens. Current evidence and expert consensus suggest HSCT in SLE as “clinical option”, in patients with active disease despite chronic immunosuppression with or without B-cell-targeted therapies [62–64].

The main indication for HSCT has become rapidly progressive diffuse SSc, where even in the biologic’s era, effective therapies capable of reversing tissue fibrosis and improving lung function are lacking [5]. Three randomized-controlled trials (RCTs) comparing autologous HSCT to intravenous Cy in SSc reported significant improvement in skin and broader disease-specific scores, with evidence for improvement of pulmonary function, skin fibrosis and quality-of-life [65–67]. A 5-year progression-free survival between 70 and 74% was reported in the multicenter European (ASTIS) and American (SCOT) trials and remained superior to monthly Cy in the control arm during the 10 years following HSCT in the ASTIS trial. The increased toxicity of HSCT in SSc compared to other indications was predominantly attributed to SSc-related cardiac dysfunction, especially related to pulmonary arterial hypertension, and Cy-induced cardiotoxicity. With improved screening procedures [68, 69], as well as Cy-sparing conditioning regimens, TRM decreased from 10% in the ASTIS trial to 6% within a multicenter EBMT prospective study [7], 3% in the SCOT trial [67], and 2.4% in the CAST study [70]. Based on these data, SSc is recommended as ‘standard indication’ for autologous HSCT and endorsed by updated recommendations of the European League Against Rheumatism [71]. Rarer indications of HSCT for rheumatic diseases with positive results from retrospective EBMT studies include ANCA-associated vasculitis [72], Takayasu arteritis [73] and Behcet’s disease [74].

## Considerations for HSCT in gastrointestinal diseases

The main area application of HSCT for gastroenterologic disease has been in inflammatory bowel disease [5], particularly Crohn’s Disease (CD) with 215 patients reported to the EBMT registry to date (Fig. 1). In addition to single-arm series [75–79], including a large EBMT retrospective study [80], one RCT, the EBMT-sponsored ASTIC trial, has reported promising results. Although the ambiguous endpoint of the ASTIC study, i.e., no evidence of active disease on endoscopy or imaging with a CD activity index <150 for at least 3 months while off all CD medications, was not achieved, data supported improvement in clinical and endoscopic measures of activity and quality-of-life, with 50% of patients showing complete endoscopic mucosal healing [81]. Accordingly, CD is suggested as “clinical option” for HSCT and the EBMT and the European Crohn’s and Colitis Organization (ECCO) provided a collaborative position paper to guide the current practice and forward development of HSCT in CD [82]. Future developments in the field include the utilization of low intensity regimens, currently investigated in the ASTIC-lite study [83], and the use of umbilical cord blood (UCB) allogeneic HSCT, which already demonstrated a 5-year long clinical, endoscopic, and histologic disease-free and drug-free survival that occurred unexpectedly without donor engraftment or risk of graft-versus-host disease (GvHD) [84].

## Considerations for allogeneic HSCT

Allogeneic HSCT is widely used to treat patients with malignant and nonmalignant hematological disorders [1]. This strategy may represent an attractive option for patients with refractory ADs, theoretically offering the advantage of complete eradication of autoreactive cells combined with the regeneration of a healthy immune system, tolerant to autoantigens [85]. In this context, the donor immune system plays a pivotal role in promoting a putative donor-versus-host alloreactivity [86, 87].

During the last decade major changes have occurred in the field of allogeneic HSCT [88–90], including the introduction of less aggressive conditioning regimens [91, 92], improved patient selection, and better supportive care, with a substantial progress in reducing GvHD because of more accurate HLA-typing and better GvHD prevention drugs, opening this procedure also to nonmalignant disorders [93–96]. Progressively the choice of donors and the sources of HSCs have enlarged, extending transplant indications to more patients [97]. Despite improved survival over time [98], allogeneic HSCT use in AD has remained rare and largely restricted to immune cytopenia, pediatric practice, and carefully selected patients affected by monogenic autoinflammatory disorders presenting with “rheumatic” phenotype [96, 99–101]. A retrospective EBMT study reported better long-term outcomes in younger patients with more recent year of transplant [85]. However, its potential to provide long-term disease control in refractory ADs paves the way for extended clinical trials to better investigate its role mainly in younger patients [85].

## Innovative cellular strategies in ADs

Alongside HSCT, innovative therapy approaches with immune-regulatory capacities such as mesenchymal stromal cells (MSC) and regulatory T cells (Tregs) have been developed over the last decades for treating severe Ads [5]. Published trials on advanced cellular therapies in this population are safe, while showing controversial beneficial effects, mainly due to a poor specificity of cell products related to a low number of true disease-relevant antigen-specific cells [102–105]. The great variability among different trials and small numbers of patients warrants further studies in this setting.

Additionally, chimeric antigen receptor (CAR)-T cells [106], one of the most promising therapy approaches for hematological malignancies, may be employed in the field of autoimmunity [107], thanks to their ability of conferring new antigen-specificities and contemporary boosting cell activation. Early data on the successful treatment of a patient with refractory SLE with CD19-targeted CAR-T cells has been recently reported [108], showing encouraging results and paving the way to this new cell strategy.

Moreover, stem cells represent an important source for potential therapeutic interventions in regenerative medicine [5]. Among them, induced pluripotent stem cells (IPS) probably resemble the most promising future approach. IPS are generated from somatic cells that have been reprogrammed by the ectopic expression of defined embryonic transcription factors (typically: Oct3/4, Sox2, c-Myc, Klf4) called “Yamanaka factors” (OSKM) [109]. They are currently under investigation for the treatment of Parkinson’s disease, macular degeneration and type I diabetes mellitus. In addition, neuronal stem cells (NSC) are increasingly evaluated as potential source for replacing damaged or lost neurons and glia [110]. Their therapeutic application already demonstrated promising results in the context of spinal cord injury in rodents [111]. Other areas of investigation include the exploitation of the inherent biologic advantages of UCB stem cells with high proliferative potential and good damage repair capacity [112], and the biologic properties, therapeutic mechanisms and clinical efficacy of MSC or MSC-derived exosomes [113, 114].

## International registries: activity of HSCT in autoimmune diseases

Two major transplant registries, the European Society for Blood and Marrow Transplantation (EBMT) and the Center for International Blood and Marrow Transplant Research (CIBMTR), capture data from many ADs patients [5].

In EBMT, quality in HSCT and cellular therapy is assured through the Joint Accreditation Committee of the International Society for Cellular Therapy and EBMT (JACIE) accreditation, which has been central to EBMT recommendations and may potentially affect the outcome of HSCT [8]. The EBMT Autoimmune Diseases Working Party (ADWP) is central to bringing together HSCT and disease specialist communities. The ADs section of the EBMT Registry is the largest database of its kind worldwide (Figs. 1, 2). As of February 2022, a total of 3789 HSCT procedures (autologous in 93% of cases) for autoimmune indications have been reported in the EBMT registry. The CIBMTR Research Database captures outcomes of patients who receive an HSCT and follows patients longitudinally until death or lost to follow-up. The CIBMTR’s study of HSCT for AID is overseen by the Nonmalignant Disease Working Committee. In the future, registry activity will continue to be essential for the further development of the field.

## Conclusions

Current evidence support HSCT as a valid treatment option in the management of selected patients with ADs, with a clear risk/benefit ratio as carefully evaluated by appropriately constituted multidisciplinary team (including transplant and disease specialists). HSCT can induce lasting remissions in patients with severe autoimmune disorders, underlying the value of these therapeutic approaches and the potential for immune reconstitution and immune resetting of T and B-cell repertoire. Center experience, accreditation, inter-specialty networking, and national socioeconomic factors are relevant for health service delivery of HSCT in ADs. New insights are coming also in the complexity and power of innovative cellular therapies. Future studies are warranted to further elucidate the mechanism of action of these cellular-based therapies, while shedding light on the underlying pathogenesis of ADs.

## Data Availability

The datasets generated for this study are available on request to the corresponding author.
